# A Paste That Will Adhere to Anything

**Published:** 1890-04

**Authors:** 


### A Paste that Will Adhere to Anything.

Professor Alex. Winchell is credited with the invention of a cement that will stick to anything. Take two ounces of clear gum arabic, one and a half ounces of fine starch, and half an ounce of white sugar. Pulverize the gum arabic, and dissolve it in as much water as the laundress would use for the quantity of starch indicated. Dissolve the starch and sugar in the gum solution. Then cook the mixture in a vessel suspended in boiling water until the starch becomes clear. The cement should be as thick as tar, and kept so. It can be kept from spoiling by dropping in a lump of gum camphor, or a little oil of cloves or sassafras. This cement is very strong, indeed, and will stick perfectly to glazed surfaces, and is good to repair broken rocks, minerals, or fossils. The addition of a small amount of sulphate of aluminum will increase the effectiveness of the paste, besides helping to prevent decomposition.—*Nat. Drug.*

